# A fully-automated paper ECG digitisation algorithm using deep learning

**DOI:** 10.1038/s41598-022-25284-1

**Published:** 2022-12-05

**Authors:** Huiyi Wu, Kiran Haresh Kumar Patel, Xinyang Li, Bowen Zhang, Christoforos Galazis, Nikesh Bajaj, Arunashis Sau, Xili Shi, Lin Sun, Yanda Tao, Harith Al-Qaysi, Lawrence Tarusan, Najira Yasmin, Natasha Grewal, Gaurika Kapoor, Jonathan W. Waks, Daniel B. Kramer, Nicholas S. Peters, Fu Siong Ng

**Affiliations:** 1 Imperial College London, National Heart & Lung Institute, London, W12 0NN UK; 2 National University of Singapore, Singapore, Singapore; 3 Department of Cardiology, Imperial College Healthcare NHS Trust, London, UK; 4 CentraleSupélec, Paris, France; 5 Harvard-Thorndike Electrophysiology Institute, Beth Israel Deaconess Medical Centre, Harvard Medical School, Boston, MA USA; 6 Cardiac Electrophysiology, National Heart and Lung Institute, Imperial College London, 4th floor, Imperial Centre for Translational and Experimental Medicine, Hammersmith Campus, Du Cane Road, London, W12 0NN UK

**Keywords:** Cardiology, Mathematics and computing, Information technology, Software

## Abstract

There is increasing focus on applying deep learning methods to electrocardiograms (ECGs), with recent studies showing that neural networks (NNs) can predict future heart failure or atrial fibrillation from the ECG alone. However, large numbers of ECGs are needed to train NNs, and many ECGs are currently only in paper format, which are not suitable for NN training. We developed a fully-automated online ECG digitisation tool to convert scanned paper ECGs into digital signals. Using automated horizontal and vertical anchor point detection, the algorithm automatically segments the ECG image into separate images for the 12 leads and a dynamical morphological algorithm is then applied to extract the signal of interest. We then validated the performance of the algorithm on 515 digital ECGs, of which 45 were printed, scanned and redigitised. The automated digitisation tool achieved 99.0% correlation between the digitised signals and the ground truth ECG (n = 515 standard 3-by-4 ECGs) after excluding ECGs with overlap of lead signals. Without exclusion, the performance of average correlation was from 90 to 97% across the leads on all 3-by-4 ECGs. There was a 97% correlation for 12-by-1 and 3-by-1 ECG formats after excluding ECGs with overlap of lead signals. Without exclusion, the average correlation of some leads in 12-by-1 ECGs was 60–70% and the average correlation of 3-by-1 ECGs achieved 80–90%. ECGs that were printed, scanned, and redigitised, our tool achieved 96% correlation with the original signals. We have developed and validated a fully-automated, user-friendly, online ECG digitisation tool. Unlike other available tools, this does not require any manual segmentation of ECG signals. Our tool can facilitate the rapid and automated digitisation of large repositories of paper ECGs to allow them to be used for deep learning projects.

## Introduction

There has been growing interest in applying machine learning to electrocardiograms (ECG). For example, variations of wavelet analysis and local binary patterns were used for extracting features from ECG, then support vector machine (SVM), k-nearest neighbour (kNN), and state-of-the-art deep neural networks were explored for arrhythmia diagnosis^1–5^. Convolutional neural networks (CNN) have also been used to predict the likelihood of paroxysmal atrial fibrillation (AF) from sinus rhythm ECGs^6–8^, screening left ventricular systolic dysfunction to identify incident heart failure^9–14^, screening hypertrophic cardiomyopathy^15–17^, and early diagnosis of valvular diseases such as aortic stenosis and mitral regurgitation^18–20^. The application of machine learning requires large volumes of ECGs in an electronic format, although, in clinical practice, they are often printed on paper and are not available in a digitised format. The practicalities of accessing and utilising large volumes of paper ECGs that have not been saved electronically can be particularly challenging. Although data repositories containing ECG data are increasingly available, the accessibility to ECGs for machine learning applications would be greatly increased with an automated digitisation tool that can rapidly convert large volumes of historical paper-based ECGs into digital signals.

A number of attempts have been made to develop 12-lead ECG digitisation tools^21–23^. For example, ECGscan^21^ was the first such application to be commercialised but requires significant user input to identify the regions of the ECG that require digitisation. Similarly, other digitisation tools^22–24^ also require manual input to ensure that the ECG leads are correctly identified by the end-user. Others have developed ECG digitisation tools to work directly on segmented single-lead ECG image^25–27^. There have been other efforts to develop automated digitisation tools that require no manual inputs, but again, those algorithms can only digitise ECGs with leads printed in a specific configuration^28^. Another approach involved applying a pre-set binary mask to obtain the region of interest, though the generalisation is limited to a single and specific layout of ECG signals^29^. In addition, ECG digitisation tools have been developed for diagnosis and monitoring cardiac disease^30^. However, there is no single method that is applicable to all paper ECG configurations and that does not require manual intervention. Some existing methods are validated using ECG parameters such as PR, QRS, RR, QT intervals, or heart rate^25–27^ rather than using a direct comparison with an original digitised version of the ECG. There is an unmet need for a user-friendly, accurate, generalisable, and fully-automated ECG digitisation tool that can be applied to paper ECGs with different configurations.

To address these limitations, we sought to develop an open-access fully-automated algorithm that can digitise 12-lead ECGs with signals printed in any standard configuration and requires no user input. We incorporate this functionality in a user-friendly interface, and we envisage that our tool will enable a large number of ECGs to be readily digitised, for machine learning purposes.

## Methods

**Figure 1:**
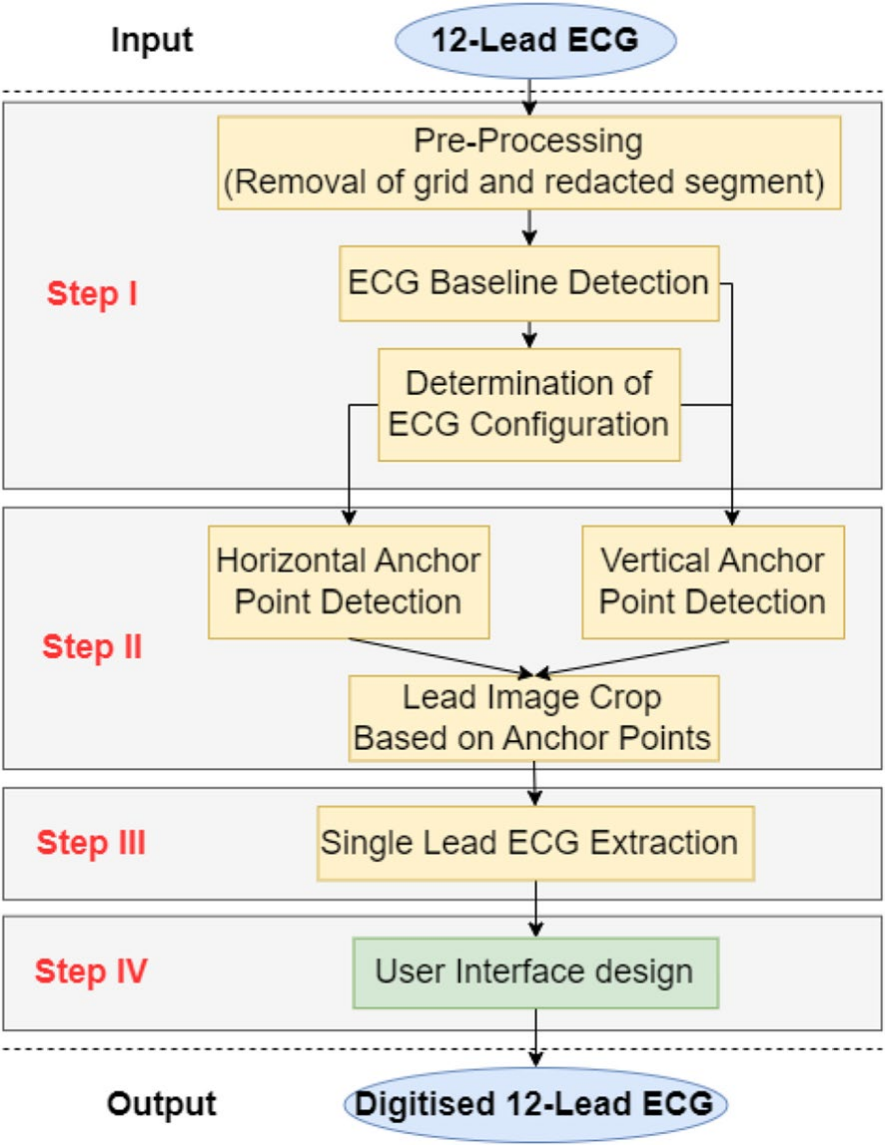
Overview of the automated ECG digitisation algorithm: Step I: The 12-lead ECG image is pre-processed to remove redacted portions of the ECG and the ECG grid. The ECG baselines are then determined to obtain the ECG configuration, aided by vertical anchor points. Step II: After determining horizontal and vertical anchor points and lead configuration, the 12-lead signals are cropped. Step III: ECG signal extraction from the single lead ECG images. Step IV: User interface design using dashboard tool.

Figure 1 outlines our automated ECG digitisation algorithm. The pseudocode of the ECG digitisation is shown in Algorithms 1–7 in Supplementary information. The paper ECG image was first pre-processed to remove any redacted regions and grids lines, and then transformed into a binary image, which enabled the ECG baselines to be subsequently detected. Once the ECG baselines were detected, vertical anchor points were used to detect the upper and lower boundary of each ECG lead signal. This step also allows the algorithm to determine the layout of the ECG leads (i.e., number of rows) on the printed ECG. Next, using lead name detection, the horizontal anchor points of each lead, i.e. left-and-right-hand boundaries of the ECG signals to be digitised, that signified their start and end, respectively, were used to crop and extract the signals in each lead of the 12-lead ECG. Finally, signals in each of the leads were digitised individually. We have developed an open-access online tool to allow users to upload scanned ECGs to extract the digital signals (http://ecg-digitisation.hh.med.ic.ac.uk:8050/) (Running speed details of the website are shown in Supplementary information). Each of these steps are described in greater detail below.

### Data source for development

Our online ECG digitisation tool was developed using 12-lead ECGs recorded in patients presenting to Imperial College London NHS Trust. These ECGs were originally printed on paper and were provided to the research team as anonymised scanned versions in Portable Document Format (PDF), and subsequently reformatted into 250 dpi Portable Network Graphics (PNG) files. These ECGs were typically in the conventional 3 $\times$ 4 lead configuration with a lead II rhythm strip. This database contained only paper ECGs, without digital ECG ground truth data.

For validation, we used anonymised 12-lead ECGs from Beth Israel Deaconess Medical Centre (BIDMC), Boston MA, USA, as PNG files in 3 $\times$ 4, 12 $\times$ 1 and 3 $\times$ 1 lead configurations to validate our digitisation tool. This second database contained both ECG images and digital ECG ground truth data. All ECGs used in the development and testing of our digitisation tool were calibrated to 1 mV = 10 mm and recorded at a paper speed of 25 mm/s.

Both the Imperial College and BIDMC provided ethics review for this project. All methods were carried in accordance with relevant guidelines and regulations. Ethical approval for collection of data used in this study was granted by Health Research Authority London Research Ethics Committee (Hampstead) (protocol number 20HH5967, REC reference 20/HRA/2467, sponsor Imperial College London). Informed consent was obtained from all subjects and/or their legal guardian(s). This study conforms to the Declaration of Helsinki.

### Step I: Determining ECG baseline and lead configuration

#### Pre-processing

In the database for development, all ECGs contained a header made up of black pixels of redacted patient information, which may adversely influence digitisation of ECG traces. For this reason, before implementation of the digitisation process, the redacted area of each ECG was automatically removed. The redacted region was black resulting in the average pixel intensity of each row of the redacted region as zero, while the average pixel intensity became a positive scalar value in regions of interest to be digitised. This enabled the redacted region to be reliably identified and removed prior to the digitisation of the ECG signals.

**Figure 2:**
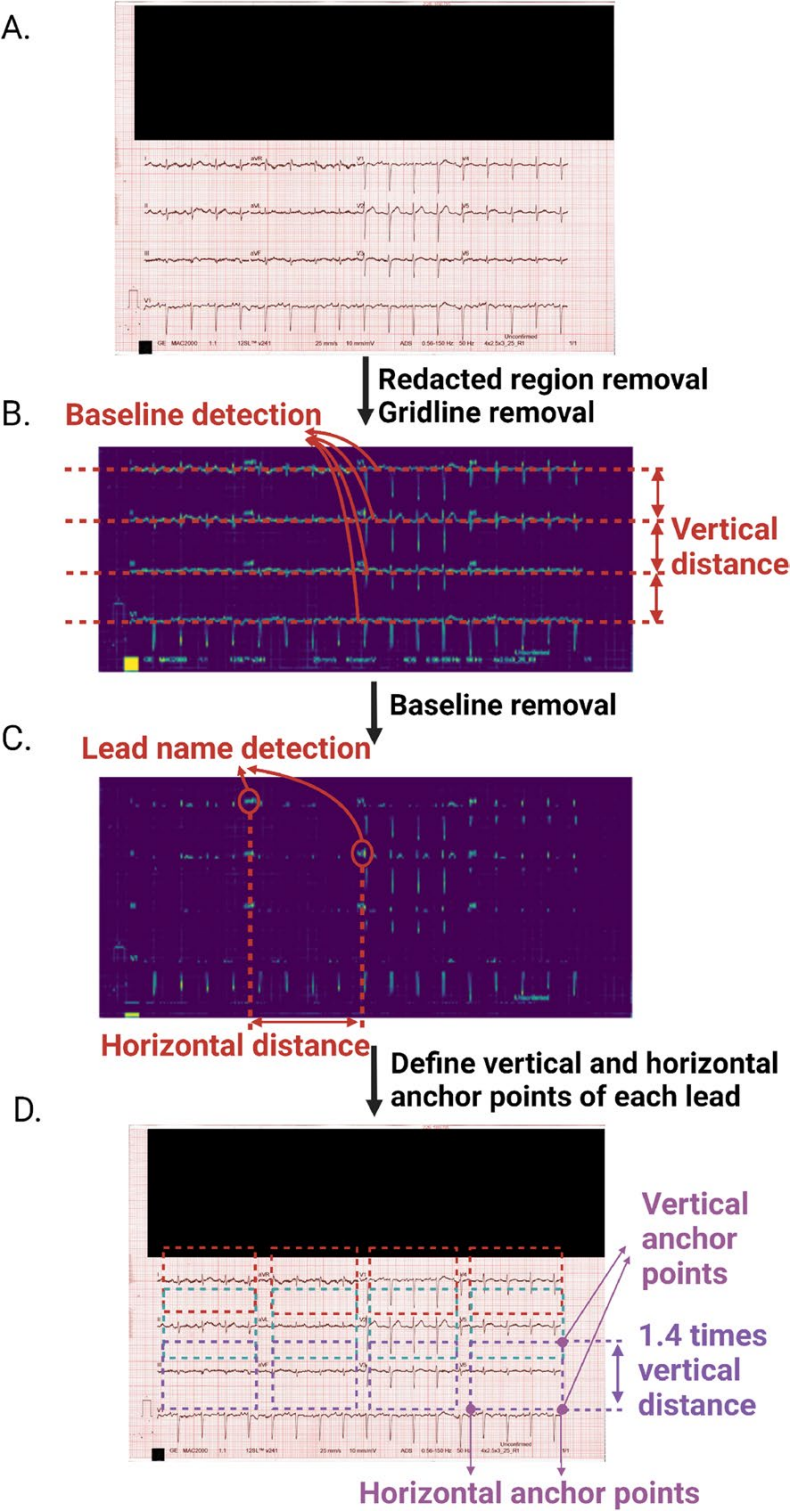
Cropping of individual ECG signal images for each lead: (**A**) The original 12-lead ECG scan with patient identifiable information redacted; (**B**) Baseline detection is used to determine the vertical distance between leads; (**C**) Lead name detection are used to determine the horizontal distance between leads; (**D**) Cropping to obtain each lead’s ECG signal. The width of the crop is the distance from end point of the lead name to the starting point of the adjacent lead name, while the height of the crop is 1.4 times of the vertical distance with the detected baseline in the middle.

ECGs are routinely printed on paper containing gridlines which were removed prior to the digitisation process. Given that ECG contained red pixels, the red channel of the image was set as 1, and the image transformed to grey-scale. A threshold of 0.94 was used to differentiate pixels that made up the ECG signal versus gridlines. Pixels $>0.94$ were discarded and those with $\le 0.94$ were taken as indicative of an ECG signal or lead name. In this way, the ECG traces and the lead name information were extracted in the binary image and the background and gridlines were eliminated. The processed binary image is shown graphically from Fig. 2A, B.

#### ECG baseline detection and ECG configuration determination

After pre-processing, the first step of the automated digitisation process required the algorithm to detect the signal baseline and determine the number of rows of ECG signals to determine the ECG configuration. We considered ECG baselines as the horizontal lines that have the highest intensities of ECG signals on the horizontal axis.

Hough transform^31^ is a coordinate transformation that converts images from Cartesian to polar axes, and has been used for computer vision feature extraction on digital images. Here, we applied Hough transform to identify the ECG baselines. In order to perform Hough transform and constrain the number of plausible solutions, two restraints were implemented to avoid inaccurate identification of the baseline. First, given that the ECG baseline is expected to be near horizontal, only lines between $-$2.5$^{\circ }$ and +2.5$^{\circ }$ around the x-axis were considered. Second, given that the baseline is expected to extend almost across the entire image, any lines less than 80% of the width of the printed ECG were discarded. In instances where there were spaces between ECG lead waveforms, the lines were merged if the inter-lead space was no greater than 15% of the total width of the image. This ensured that the ECG signals of adjacent leads remained independent and were not combined in the digitisation process. This method also helped to determine the number of baselines on the printed ECG, and in conjunction with the vertical anchor point detection below, provided information on the lead configuration.

### Step II: Automated anchor point detection

#### Vertical anchor point detection

Just as baseline detection was used to determine vertical anchor points to identify ECG signals in space, vertical anchor points were used to determine the upper and lower boundaries of the signals in each ECG lead to identify the signals to be digitised. The vertical cropping length is presented in Fig. 2B. The upper and lower boundaries were defined as 0.7 times the distance between two neighbouring ECG signals (in the horizontal plane) above and below the ECG baseline, respectively.

#### Horizontal anchor point detection

Horizontal anchor points were used to determine the left- and right-hand boundaries of the ECG signals to be digitised, that signified their start and end, respectively. The lead name and the start of the subsequent ECG signal in the horizontal plane constituted the start and end of the ECG signal to be digitised. The maximum horizontal distance encompassing the ECG signal in other leads in the same ECG was used to define the right-hand boundary for leads on the far right of the image that had no right-hand boundary.

Our text recognition model was unable to detect lead names when these were in close proximity to the ECG baseline. In these instances, ECG baselines were removed to enable the digitisation tool to identify the lead names. Additionally, morphological dilation and erosion were applied to the image to enhance the distinguishability of the lead names to surrounding signals. Thus, it enabled the text recognition model to identify these cases more easily. Dilation is an iterative region-growing algorithm that thickens the lines and erosion is an iterative region-shrinking algorithm that thins the lines, thereby making any objects of interest more readily identifiable by automated processes. All objects of interest in the image were filtered in this way to exclude those with width-height-ratio $>5$ and those with a width or height $<5$ pixels or $>500$ pixels.

Thereafter, a trained text character recognition deep learning model^32^ was used to specifically detect lead names amongst the other filtered objects. The input for the model comprised the 12-lead ECG binary image and 12 ground truth lead name text strings (‘I’, ‘II’, ‘III’, ‘avr’, ‘avl’, ‘avf’, ‘v1’, ‘v2’, ‘v3’, ‘v4’, ‘v5’, ‘v6’). The output constituted any texts detected by the model, the corresponding bounding box for the text, and the confidence score. Thresholds of confidence scores were set to detect lead names such that the identification of one of the text strings would result in a confidence score exceeding the threshold. In this way, lead name objects, the position, height and width information of the lead name objects were identified for their implementation as horizontal anchor points. The process of obtaining horizontal distance from lead name detection is presented in Fig. 2C. In instances when some lead name detection was unsuccessful, horizontal anchor points were determined based on the distance between other lead names that were successfully identified in the same ECG. ECG segments for each lead were cropped after successfully identifying the horizontal and vertical anchor points, and is shown in Fig. 2D.

### Step III: Single lead ECG extraction

**Figure 3:**
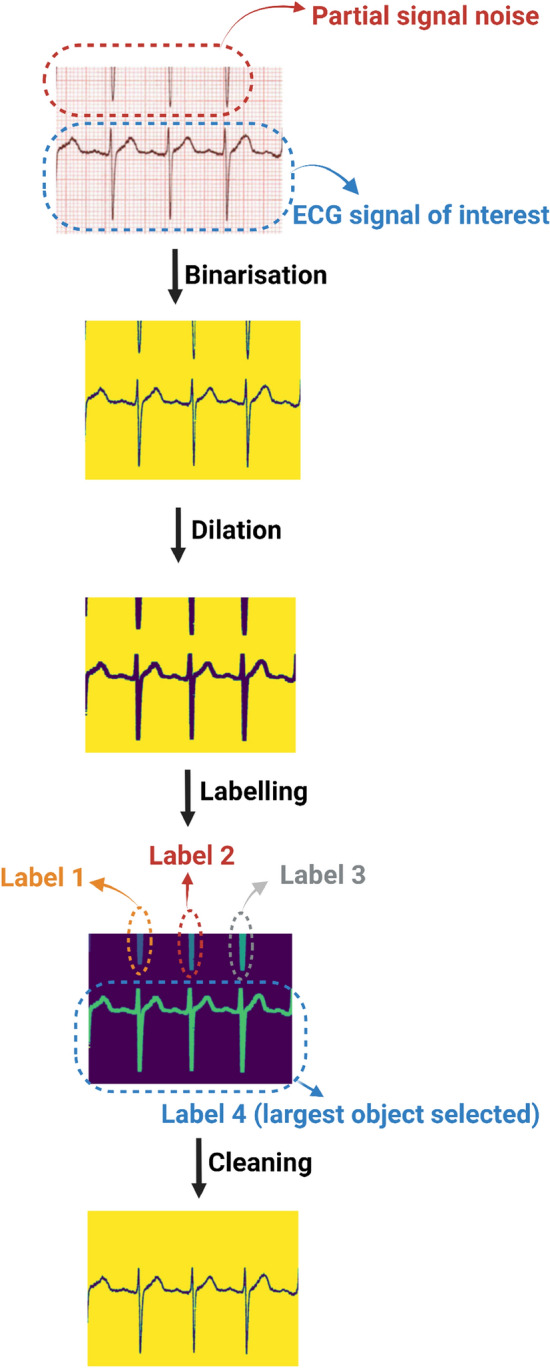
The cleaning process of a cropped ECG image. Following the cropping of the region of interest, a dilation process connects the possible breaking points horizontally to obtaining the full ECG signal. Thereafter, the labelling process identifies the largest object as the signal of interest. Finally, artefacts within the cropped image are removed to retain the signal of interest.

The extraction of the ECG signals from the cropped image required removal of “salt-and-pepper” noise, that comprises sparse white and black pixels, as well as any partial ECG signals from other leads. The latter is particularly true for large amplitude ECG traces that would encroach the cropped images of neighbouring leads as shown in Fig. 3. To do this, first we used image dilation to connect any discontinuities in the ECG signal of interest which also prevented any spurious connections with noise or neighbouring signals. Thereafter, we considered the largest detectable object in the image as the ECG signal of interest and all other objects as artefacts. This process is presented in Fig. 3 which demonstrates this method retains the signal of interest and removes other objects contained within the cropped image.

The next step involved converting the extracted ECG binary image into a one-dimension digital ECG signal. The ECG signal in the binary image comprises a set of pixels with x (time) and y (voltage) coordinates, calibrated at 25 mm/s and 10 mm/mv. For any given point in time (x-axis), several pixels may make up the corresponding amplitude. Given that the digital ECG signal can only have a single y-coordinate for each x-coordinate, we used the median amplitude pixel (y-axis) in the binary image to reconstruct the digital ECG signal. This generated a digital ECG signal with x and y coordinates in pixel units. In order to ascribe time and voltage values to the digital ECG signal, we determined time and voltage resolutions using the rhythm (or longest signal) strip in each ECG. Given that a standard 12-lead ECG duration is 10 s, the time resolution was calculated as the 10 s divided by the number of pixels in the x-axis. The voltage-time resolution ratio is standard at 0.1 mV/40 ms = 0.0025 mV/ms which enabled the voltage resolution of the signals to be determined by multiplying time resolution and voltage-time resolution ratio (0.0025 mV/ms). In this way, the time of the digital ECG signal was calculated as the number of pixels in the x-axis multiplied by time resolution, and the amplitude as the number of pixels on y-axis multiplied by voltage resolution.

### Step IV: Dashboard online tool development

We developed the online tool with Python dash plotly. The following steps provides step-by-step instructions for the end-user to use the online tool. First, the users are required to scan and upload an ECG image. Users are reminded to fully redact and anonymise all confidential or patient-identifiable data. The image is read by the Python method “cv2.imread” and can support any image format that is supported by “cv2.imread”. After uploading the image, it is displayed with a fixed height 600 pixels (px). Next, a dropdown bar provides options to visualise each digitised ECG signal with the option of changing the resolution by magnifying or minimising the image. The digitised ECG can be downloaded into a spreadsheet containing 13 columns, with the first column providing data for the time axis and remaining 12 columns are ECG signal data in voltage.

### Statistical analyses

We validated our tool using Pearson’s correlation and root mean squared error (RMSE) to determine the association between ground truth ECG signals and digitised ECG signals generated by our digitisation tool. The validation was conducted on the independent database obtained from BIDMC. Pearson’s correlation and Root Mean Squared Error (RMSE) were performed using Python (“scipy.stat.pearsonr” for Pearson’s correlation and “sklearn.metrics.mean_squared_error” for RMSE). $P<0.001$ was considered significant.

## Result

We validated our digitisation tool using three independent validation tests. The digitisation tool was developed using a database of paper ECGs. Consequently, parameters (QRS duration, PR, QT and RR intervals) from these ECGs were the only method for validating our tool. To obtain more accurate validation, we performed validation using an external ECG database from BIDMC containing digital ECGs.

**Figure 4:**
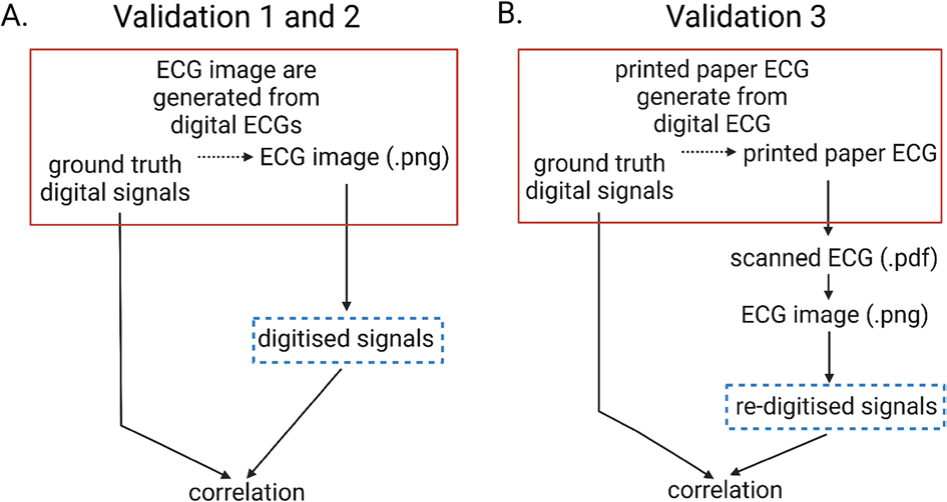
Validation of ECG digitisation tool. Text in red boxes represents input data. Digitised signals generated by our digitisation tool are indicated in blue dashed boxes (**A**) Validation 1 and 2: comparison of digitised ECG traces with ground truth digital signals; (**B**) Validation 3: comparison of digitised ECG traces from digital ECGs that were printed, scanned and re-digitised, with ground truth digital signals.

### Validation 1: 3 $\times$ 4 ECGs

**Figure 5:**
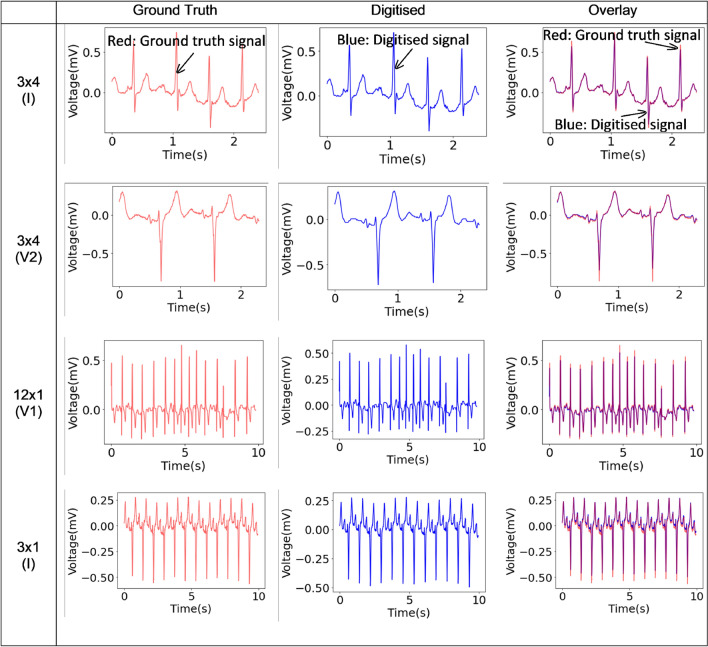
Digitisation results and ground truth comparison: ground truth original digital ECG signals (left) and digitised signals from images (centre) shown together with the overlay of both traces (right), for multiple ECG configurations. Ground truth signal is shown as red and digitised signal is shown as blue, the overlay of comparison plot shows two coloured signals overlapped. The overlay shows excellent correlation between the ground truth and digitised signals.

This validation was performed with acquired digital and printed ECGs (Fig. 4A). There are overall 930 standard 3 $\times$ 4 ECG images that are validated. 7 3 $\times$ 4 ECG images failed in lead name detection, which are shown in the Supplementary Fig. S1. The average correlation and RMSE performance of the remaining 923 3 $\times$ 4 ECG images is shown in Table 1. The performance of average correlation is from 90 to 97% across the leads. 515 3 $\times$ 4 ECG images without overlap of lead signals are selected for validation from the 923 3 $\times$ 4 ECG images by a cardiologist to eliminate the effect of lead signal overlapping. The performance of correlation and RMSE between 515 digitised ECGs and ground truth ECG signals in a 3 $\times$ 4 configuration are shown in Table 2. The average correlation value was consistently $>99\%$ across all leads ($p<0.001$), and the average RMSE were consistently 0.04 mV ($p<0.001$). Examples of this validation is presented in Fig. 5, in which the red line represents the ground truth and the blue line the digitised result.

### Validation 2: 12 $\times$ 1 and 3 $\times$ 1 ECGs

Next, we performed validation on 310 ECGs in 12 $\times$ 1 and 91 ECGs in 3 $\times$ 1 lead configurations (Fig. 4A). There were 2 12 $\times$ 1 ECG images and 4 3 $\times$ 1 ECG images that failed in lead name detection (Supplementary Figs. S2 and S3). The average correlation and RMSE performance of the remaining 308 12 $\times$ 1 ECG images and 87 3 $\times$ 1 ECG images are shown in Tables 3 and 4. Some leads’ average correlation performance drops between 60 and 70% due to severe overlapping of ECG signals in 12 by 1 ECG configurations, and the average correlation performance of 3 by 1 ECG signals achieved 80–90%. Similarly, to get rid of the overlapping images, 45 12 $\times$ 1 ECG images were selected from 308 12 $\times$ 1 ECG images, and 51 3 $\times$ 1 ECG images were selected from 87 3 $\times$ 1 ECG images by a cardiologist for validation. The correlations between digitised ECGs and the ground truth signals of 45 12 $\times$ 1 and 51 3 $\times$ 1 thresholded ECGs are shown in Tables 5 and 6 respectively, and consistently exceeded 97% in all leads ($p<0.001$), and the average RMSE were consistently 0.04 mV ($p<0.001$). Examples of digitised and ground truth ECG traces are shown in Fig. 5.

### Validation 3: ECG images and prints

Finally, we validated our digitisation tool against 45 images of printed ECGs in a 3 $\times$ 4 configuration. This validation process is shown in Fig. 4B. For this validation process, we printed each ECG image and re-scanned it to generate an ECG in PDF format. This was then transformed into PNG-image, to which our digitisation tool was applied. Digitisation was unsuccessful in one ECG in which lead name could not be detected, although digitisation was successful in its equivalent digital copy, suggesting that the resolution of the printed ECG was of poor quality. The correlation between the digitised and remaining 44 scanned ECGs are shown in Table 7. The average correlation value between digitised and validation ECGs was 96% across all leads ($p<0.001$), and the average RMSE were consistently 0.05 mV ($p<0.001$). These results demonstrate that our digitisation tool can be successfully generalized to both ECG images and ECG paper scans.

**Table 1:** Correlation and root mean squared error (RMSE) statistics of the digitised results from 923 standard 3 by 4 ECG images and the ground truth digital ECG before image thresholding (validation 1).

Lead name	Correlation				Root mean squared error			
	Average	SD	*p*-value	95% confidence interval	Average	SD	*p*-value	95% confidence interval
I	0.909	0.196	$<0.001$	0.896–0.921	0.098	0.154	$<0.001$	0.088–0.108
II	0.974	0.085	$<0.001$	0.968–0.979	0.052	0.051	$<0.001$	0.049–0.056
V1	0.966	0.088	$<0.001$	0.961–0.972	0.048	0.057	$<0.001$	0.045–0.056
V2	0.952	0.134	$<0.001$	0.943–0.961	0.071	0.113	$<0.001$	0.064–0.078
V3	0.934	0.160	$<0.001$	0.924–0.945	0.090	0.133	$<0.001$	0.081–0.098
V4	0.928	0.163	$<0.001$	0.917–0.938	0.090	0.102	$<0.001$	0.083–0.096
V5	0.946	0.133	$<0.001$	0.937–0.955	0.078	0.077	$<0.001$	0.073–0.083
V6	0.973	0.092	$<0.001$	0.967–0.979	0.062	0.064	$<0.001$	0.058–0.066

**Table 2:** Correlation and root mean squared error (RMSE) statistics of the digitised results from 515 standard 3 by 4 ECG images and the ground truth digital ECGs (validation 1).

Lead name	Correlation				Root mean squared error			
	Average	SD	*p*-value	95% confidence interval	Average	SD	*p*-value	95% confidence interval
I	0.988	0.014	$<0.001$	0.986–0.989	0.043	0.026	$<0.001$	0.041–0.045
II	0.988	0.025	$<0.001$	0.986–0.990	0.043	0.033	$<0.001$	0.040–0.046
V1	0.991	0.018	$<0.001$	0.989–0.992	0.033	0.027	$<0.001$	0.031–0.036
V2	0.991	0.016	$<0.001$	0.989–0.992	0.040	0.031	$<0.001$	0.037–0.043
V3	0.992	0.007	$<0.001$	0.991–0.992	0.044	0.030	$<0.001$	0.041–0.046
V4	0.991	0.008	$<0.001$	0.990–0.992	0.052	0.041	$<0.001$	0.048–0.055
V5	0.991	0.007	$<0.001$	0.990–0.991	0.050	0.038	$<0.001$	0.047–0.053
V6	0.991	0.009	$<0.001$	0.991–0.992	0.049	0.045	$<0.001$	0.045–0.053

**Table 3:** Correlation and root mean squared error (RMSE) statistics of the digitised results from selected 308 12 by 1 ECG images and the ground truth digital ECGs before image thresholding (validation 2).

Lead name	Correlation				Root mean squared error			
	Average	SD	*p*-value	95% confidence interval	Average	SD	*p*-value	95% confidence interval
I	0.894	0.209	$<0.001$	0.871–0.918	0.073	0.078	$<0.001$	0.064–0.082
II	0.933	0.197	$<0.001$	0.911–0.955	0.063	0.085	$<0.001$	0.054–0.073
V1	0.880	0.261	$<0.001$	0.850–0.909	0.086	0.184	$<0.001$	0.065–0.106
V2	0.772	0.315	$<0.001$	0.737–0.807	0.139	0.184	$<0.001$	0.119–0.160
V3	0.660	0.296	$<0.001$	0.627–0.693	0.174	0.157	$<0.001$	0.156–0.187
V4	0.638	0.287	$<0.001$	0.606–0.670	0.168	0.125	$<0.001$	0.154–0.182
V5	0.724	0.295	$<0.001$	0.690–0.757	0.139	0.116	$<0.001$	0.126–0.152
V6	0.822	0.302	$<0.001$	0.788–0.856	0.100	0.109	$<0.001$	0.088–0.112

**Table 4:** Correlation and root mean squared error (RMSE) statistics of the digitised results from 87 3 by 1 ECG images and the ground truth digital ECGs before image thresholding (validation 2).

Lead name	Correlation				Root mean squared error			
	Average	SD	*p*-value	95% confidence interval	Average	SD	*p*-value	95% confidence interval
I	0.863	0.201	$<0.001$	0.820–0.906	0.076	0.070	$<0.001$	0.061–0.091
II	0.907	0.206	$<0.001$	0.863–0.950	0.067	0.066	$<0.001$	0.053–0.081
V1	0.898	0.235	$<0.001$	0.848–0.948	0.068	0.095	$<0.001$	0.048–0.088

**Table 5:** Correlation and root mean squared error (RMSE) statistics of the digitised results from selected 45 12 by 1 ECG images and the ground truth digital ECGs (validation 2).

Lead name	Correlation				Root mean squared error			
	Average	SD	*p*-value	95% confidence interval	Average	SD	*p*-value	95% confidence interval
I	0.963	0.015	$<0.001$	0.959–0.968	0.039	0.012	$<0.001$	0.035–0.043
II	0.979	0.011	$<0.001$	0.975–0.982	0.033	0.016	$<0.001$	0.029–0.038
V1	0.981	0.013	$<0.001$	0.977–0.985	0.029	0.011	$<0.001$	0.026–0.032
V2	0.987	0.015	$<0.001$	0.982–0.991	0.033	0.016	$<0.001$	0.028–0.038
V3	0.973	0.056	$<0.001$	0.960–0.990	0.037	0.024	$<0.001$	0.030–0.044
V4	0.973	0.052	$<0.001$	0.957–0.988	0.040	0.025	$<0.001$	0.032–0.047
V5	0.986	0.013	$<0.001$	0.983–0.990	0.037	0.024	$<0.001$	0.031–0.042
V6	0.991	0.006	$<0.001$	0.990–0.993	0.034	0.015	$<0.001$	0.029–0.038

**Table 6:** Correlation and root mean squared error (RMSE) statistics of the digitised results from selected 51 3 by 1 ECG images and the ground truth digital ECGs (validation 2).

Lead name	Correlation				Root mean squared error			
	Average	SD	*p*-value	95% confidence interval	Average	SD	*p*-value	95% confidence interval
I	0.942	0.027	$<0.001$	0.935–0.950	0.049	0.017	$<0.001$	0.045–0.054
II	0.971	0.015	$<0.001$	0.967–0.975	0.045	0.023	$<0.001$	0.038–0.051
V1	0.988	0.009	$<0.001$	0.985–0.990	0.031	0.011	$<0.001$	0.028–0.034

**Table 7:** Correlation and root mean squared error (RMSE) statistics of the digitised results from 44 printed and scanned paper ECGs and the ground truth digital ECGs (validation 3).

Lead name	Correlation				Root l			
	Average	SD	*p*-value	95% confidence interval	Average	SD	*p*-value	95% confidence interval
I	0.968	0.016	$<0.001$	0.962–0.973	0.968	0.016	$<0.001$	0.962–0.973
II	0.972	0.014	$<0.001$	0.967–0.976	0.968	0.016	$<0.001$	0.962–0.973
V1	0.977	0.013	$<0.001$	0.973–0.981	0.968	0.016	$<0.001$	0.962–0.973
V2	0.973	0.025	$<0.001$	0.965–0.980	0.968	0.016	$<0.001$	0.962–0.973
V3	0.969	0.042	$<0.001$	0.957–0.982	0.968	0.016	$<0.001$	0.962–0.973
V4	0.974	0.012	$<0.001$	0.971–0.978	0.968	0.016	$<0.001$	0.962–0.973
V5	0.973	0.013	$<0.001$	0.969–0.977	0.968	0.016	$<0.001$	0.962–0.973
V6	0.974	0.014	$<0.001$	0.970–0.978	0.968	0.016	$<0.001$	0.962–0.973

## Discussion

We have developed a robust and user-friendly online ECG digitisation interface that lends itself to the digitisation of large numbers of paper ECG. Its main advantage is that it is fully-automated and can be readily applied to all printed ECGs irrespective of the lead configuration. Validation on an external database of digital ECGs showed 99.0% correlation and average 0.04 mV RMSE on 8 ECG leads in a 3 by 4 configuration after excluding the ECG images with lead signal overlap. Without this thresholding, it achieved 90–97% average correlation across the leads. In addition, we show that the software can digitise ECG signals from leads arranged in a number of configurations from printed and scanned ECGs. The average correlation of 12 by 1 ECG signals dropped to 60–70% in some leads due to the overlapping of the lead signals. However, it still achieved 97% average correlation in 12 by 1 and 3 by 1 ECG configurations after excluding the ECG images with overlapping signals.

The first step of the digitisation process required the algorithm to detect the lead configuration of the printed ECG using horizontal and vertical anchors to facilitate cropping of each lead in turn. Other digitisation tools^28^ developed a similar interface using a line detection algorithm for horizontal and vertical anchor point detection that functions with ECGs printed in a 6 $\times$ 2 configuration. Although our tool adopted a similar method for vertical anchor detection, we also applied a deep learning-based text recognition model for lead name detection for horizontal anchor point detection. This has the advantage of allowing the software to extract data from any configuration of ECG. Although horizontal anchor points may be identified by dividing the ECG image in half, this approach may not be accurate in ECG configurations where the leads are not equidistant and would only work for 6 $\times$ 2 ECG configurations. Other digitisation tools also require manual labelling of anchor points^21–24,29^ and restricted in their application by ECG configurations. They are also user-dependent, requiring manual selection of each lead prior to the digitisation process. By contrast, our digitisation tool can be utilised on a ECGs of different configurations and requires no manual inputs prior to the digitisation process. We envisage that this will aid its application in clinical and non-clinical settings to enable larger volumes of printed ECGs to be digitised in a shorter timescale.

Following lead detection and cropping of individual leads, our digitisation tool provides an efficient method for ECG signal extraction. Similar to other digitisation interfaces^28^, we apply connectivity algorithms to label and remove small objects. However, the other existing digitisation methods cannot remove all non-ECG artefacts or partial ECG signals from other leads, and this necessitates other processes, such as an iterative process to select pixels from left to right of the image. Although this methodology enables ECG extraction, it can be a complex and time-consuming process. By contrast, we utilised a dynamic morphological method to connect any discontinuities in the ECG signal prior to identifying the largest labelled object as the ECG signal of interest. This effectively eliminates noise without the need for further computational processing.

Traditionally, many existing ECG digitisation tools require manual segmentation, removal of gridlines, and processing to extract digital signals. Ravichandran et al.^22^ and Lobodzinski et al.^33^ have applied optical character recognition to scan and reference printed text with a pre-defined character template database, or to store the demographic data. Apart from the traditional methods, others used end-to-end deep learning technique for ECG digitisation^34^. However, their techniques are limited on the generalisability to different ECG image databases, especially with different configurations.

The motivation for developing our tool was to enable users to generate large volumes of digital ECGs from their paper, image, or scanned counterparts quickly and easily. We envisage that this will be particularly useful for individuals that wish to use ECGs in machine learning applications. Although this can be achieved without digitising ECGs, for example with paper ECGs or their images^30^, any outputs from these processes is inherently determined by the quality of the input. By contrast, our tool digitises paper ECGs with different configurations and thereby generates standardised inputs for machine learning algorithms.

Overall, our digitisation tool has the following advantages:

1. It is fully-automated without the need for manual user input of single lead signal segmentation.

2. Text-recognition-based lead name detection makes our digitisation tool generalisable on different configurations of ECG images, or paper-based ECG scans.

3. An efficient ECG extraction algorithm enables swift digitisation at the point of need.

4. The Pearson’s correlation and RMSE of ground truth digital ECG and digitised ECG waveform is a robust way of validation for ECG digitisation tool.

Although our method accurately extracts ECG signals, there are conditions in which we expect that the tool may not perform as desired. The limitations are listed as below:

1. Our text recognition model was trained on generic images and therefore may not always recognize lead names on printed ECGs. For instance, the tool may not consistently distinguish between the leads I, II and III accurately particularly if these are obscured by large voltage ECG signals. Lead name detection may be inaccurate in ECGs that are pixelated and of low resolution (Supplementary Figs. S1–S3).

2. Similarly, signal extraction may not be accurate in instances where there are overlapping ECG traces, particularly as shown in Table 3. We intend to apply deep neural networks (DNN) to address the limitations that would obviate the need for manual annotation of leads and serve to improve the out-of-distribution detection.

Comparisons of our digitisation tool with other existing tools are summarised in Table 8. Our digitisation tool compares favourably with these, and notably can discriminate different lead configurations.

**Table 8:** Comparison of different ECG digitisation tools: Existing digitisation tools are specific ECG configurations or do not detect ECG anchor points by automated methods.

Configurations	Badilini^21^	Mishra^24^	Isabel^29^	Ravichandran^22^	Baydoun^28^	Fortune^23^	Our model
Automatic ECG anchor point selection					$\checkmark$		$\checkmark$
6 by 2 ECG	–	–	–	–	$\checkmark$	-	$\checkmark$
3 by 4 ECG	–	$\checkmark$	$\checkmark$	$\checkmark$	–	$\checkmark$	$\checkmark$
12 by 1 ECG	$\checkmark$	–	–	–	–	–	$\checkmark$
3 by 1 ECG	–	–	–	–	–	–	$\checkmark$

By contrast, the automated tool presented here in can digitise ECGs of any configuration. (‘–’ suggests that it is unclear if the algorithm can process ECG configuration).

## Conclusion

We have developed a validated, fully-automated, user-friendly online 12-lead ECG digitisation tool that demonstrates a high degree of accuracy and reliability amongst external validation datasets. It consists of multiple logic-based modules and a sophisticated text character recognition deep learning model that enables its application to all common configurations of ECGs in different clinical settings. Furthermore, it can be utilised on printed and/or scanned ECGs and thereby enables large-scale digitisation of paper ECGs without any user-input.

## Supplementary Information

Supplementary Information.

## Data Availability

The data that support the findings of this study are available from BIDMC and Imperial College Healthcare NHS trust but restrictions apply to the availability of these data, which were used under license for the current study, and so are not publicly available. Data are however available from the authors upon reasonable request and with permission of BIDMC and Imperial College Healthcare NHS trust.
