# Supplementary material for: A fully-automated paper ECG digitisation algorithm using deep learning
Source: Sci Rep. 2022 Dec 5;12:20963. doi: 10.1038/s41598-022-25284-1 (PMC9722713; doi:10.1038/s41598-022-25284-1)

Running speed details of the website: This website is currently deployed on system with limited available resources (4v CPU, No GPU support), but will be scaled up in the future. After uploading the image, please wait for a few minutes for the digitised results to appear. Some example ECG images on the website can be clicked to download for testing.

The source code will be shared on GitHub in the future.

Pseudocode Algorithm 1.

Algorithm 1 Digitisation Overview

Input: x : Matrix $N \times M \times 3$, where $N > 0$ and $M > 0$
Output: $lead\_signals$: Matrix $K \times L$, where $K > 0$ and $L > 0$

```
x ← redacted_removal(x)                                                              ▷ Algorithm 2
x ← grid_removal(x)                                                                  ▷ Algorithm 3
baselines ← baseline_detection(x)                                                    ▷ Algorithm 4
anchor_points, vertical_distance, horizontal_distance ← detect_anchor_points(x, baselines)   ▷ Algorithm 5
x ← crop_lead_regions(x, anchor_points, vertical_distance, horizontal_distance)      ▷ Algorithm 6
lead_signals ← extract_ecg_signal(x)                                                 ▷ Algorithm 7

return lead_signals
```

Pseudocode Algorithm 2.

Algorithm 2 Redacted region removal that is marked as black pixels

Input: x : Matrix $N \times M \times 3$, where $N > 0$ and $M > 0$
Output: y : Matrix $K \times M \times 3$, where $N \geq K > 0$ and $M > 0$

```
non_zero_percentage ← 0.5
upper_y ← find_first_non_zero_row(x, non_zero_percentage)
lower_y ← find_first_zero_row(x[upper_y + 1 :], non_zero_percentage)
y ← crop_y_bounds(x, upper_row, lower_row)

return y
```

Pseudocode Algorithm 3.

Algorithm 3 Grid removal and image thresholding

Input: x : Matrix $N \times M \times 3$, where $N > 0$ and $M > 0$
Output: y : Matrix $N \times M$, where $N > 0$ and $M > 0$

```
x ← normalise(x)
x ← set_red_channel_to(x, 1)
x ← transform_to_grayscale(x)
y ← image_threshold(x, threshold = 0.94)        ▷ Pixels above threshold are set to 1, otherwise to 0

return y
```

Pseudocode Algorithm 4.

Algorithm 4 ECG baseline detection

Input: x : Matrix $N \times M \times 3$, where $N > 0$ and $M > 0$
Output: $hough\_lines$: Matrix $K \times 4$, where $K > 0$

$hough\_lines \leftarrow horizontal\_hough\_line\_transform(x)$
$hough\_lines \leftarrow merge\_close\_horizontal\_lines(hough\_lines)$
$hough\_lines \leftarrow take\_average\_close\_lines(hough\_lines)$

return $hough\_lines$

Pseudocode Algorithm 5.

Algorithm 5 Horizontal and vertical anchor point detection and estimating position of missing anchors

Input: x : Matrix $N \times M \times 3$, where $N > 0$ and $M > 0$
Input: $baselines$: Matrix $K \times 4$, where $K > 0$
Output: $anchor\_points$: Matrix $L \times 2$, where $L > 0$
Output: $vertical\_distance$: Scalar
Output: $horizontal\_distance$: Scalar

# Vertical Anchor Point Detection
$vertical\_distance \leftarrow get\_average\_of\_consecutive\_lines(baselines)$ ▷ Consecutive lines are determined if the distance between a pair exceeds x1.5 of the minimum distance found

# Horizontal Anchor Point Detection
$mask\_lines \leftarrow dilate\_lines\_vertically(baselines)$
$masked\_x \leftarrow mask\_line\_regions\_from\_image(x, mask\_lines)$ ▷ Sets masked pixels to 0
$masked\_x \leftarrow morphological\_dilation\_erosion(masked\_x)$
$masked\_x \leftarrow remove\_large\_objects(masked\_x)$
$text\_points \leftarrow identify\_text\_positions\_from\_neural\_network(masked\_x)$
$text\_points \leftarrow select\_valid\_lead\_names(text\_points)$
$horizontal\_distance \leftarrow get\_average\_consecutive\_point\_distance(text\_points)$ ▷ Consecutive points are determined by lead name, and spatial position

$anchor\_points \leftarrow determine\_anchor\_points\_on\_grid(baselines, text\_points, vertical\_distance, horizontal\_distance)$
$anchor\_points \leftarrow estimate\_missing\_anchor\_points(anchor\_points)$

return $anchor\_points, vertical\_distance, horizontal\_distance$

Pseudocode Algorithm 6.

Algorithm 6 Crop lead regions in the image

Input: x : Matrix $N \times M \times 3$, where $N > 0$ and $M > 0$
Input: $anchor\_points$: Matrix $L \times 2$, where $L > 0$
Input: $vertical\_distance$: Scalar
Input: $horizontal\_distance$: Scalar
Output: $cropped\_images$: Matrix $X \times Y \times L$, where $X > 0, Y > 0$ and $L > 0$

for each $anchor\_points, i$ **do**
  $bounding\_box \leftarrow get\_bounding\_region(i, vertical\_distance, horizontal\_distance)$
  $images[i] \leftarrow crop\_image(x, bounding\_box)$
end for

return $images$

Pseudocode Algorithm 7.

Algorithm 7 Extract digital ECG signal from cropped regions

Input: *cropped_images* : Matrix $X \times Y \times L$, where $X > 0, Y > 0$ and $L > 0$
Output: *lead_signals* : Matrix $N \times L$, where $N > 0$ and $L > 0$

```
voltage_resolution ← 0.01                                        ▷ In mV
time_resolution ← 40                                             ▷ In ms
for each cropped_images, i do
    i ← morphological_dilation(i)
    labels ← label_unique_segments(i)
    i ← select_largest_label(labels)
    signal ← get_median_y_amplitude(i)                           ▷ Generates a 1D array
    signal ← signal × voltage_resolution × get_y_size(i)
    lead_signals[i] ← interpolate(signal, get_x_size(i) × time_resolution)
end for

return lead_signals
```

Figure S1. Failed detected 3 by 4 ECG images from 930 3 by 4 ECG images (Failure Reason: lead name detection failure)

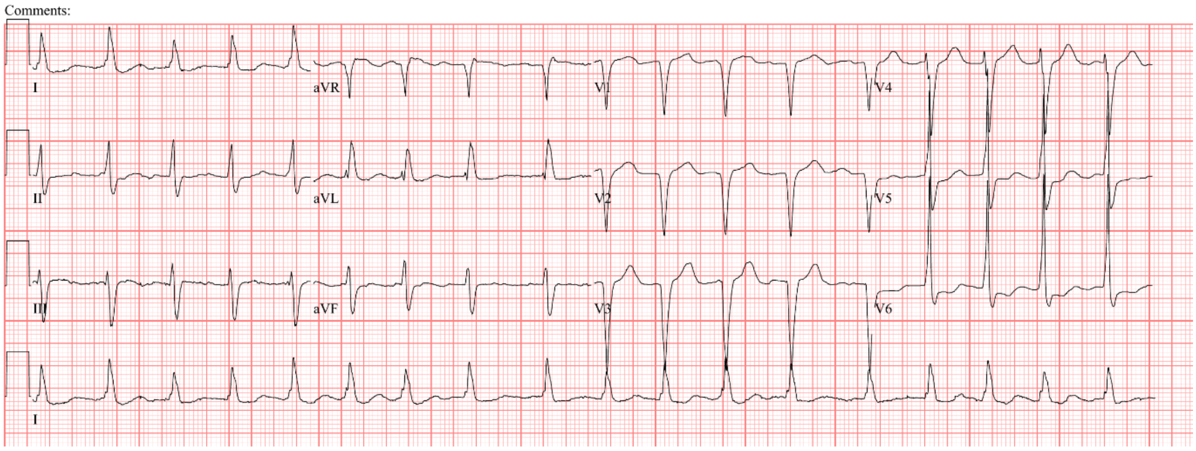

RESEARCH?: REDACTED

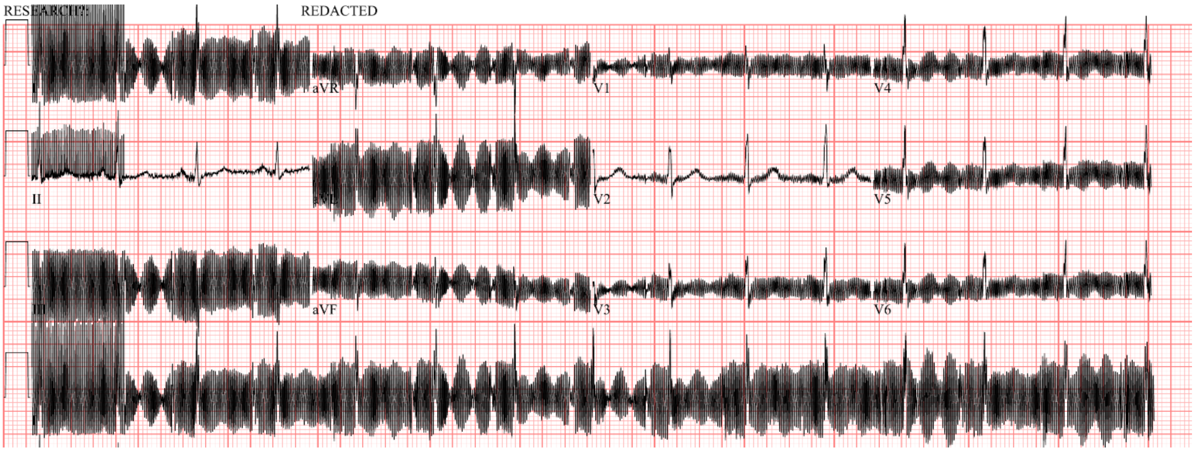

Comments:WEAKNESS USER NAME:

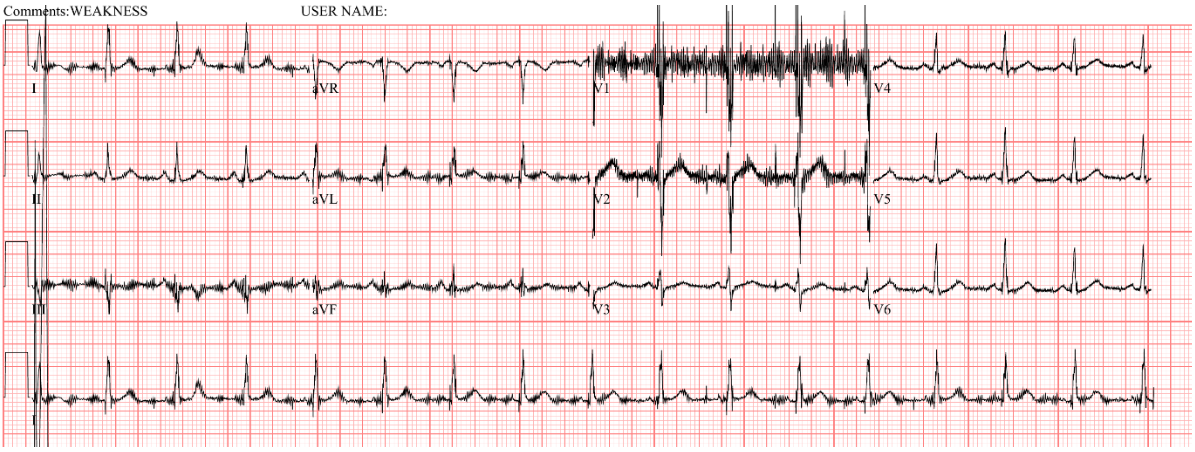

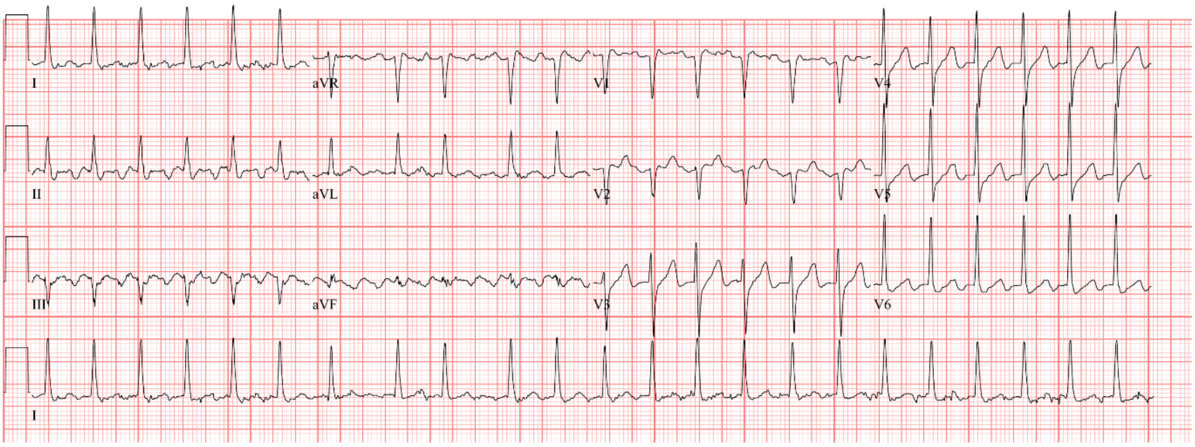

Comments:HYPERTENSION Operator I:

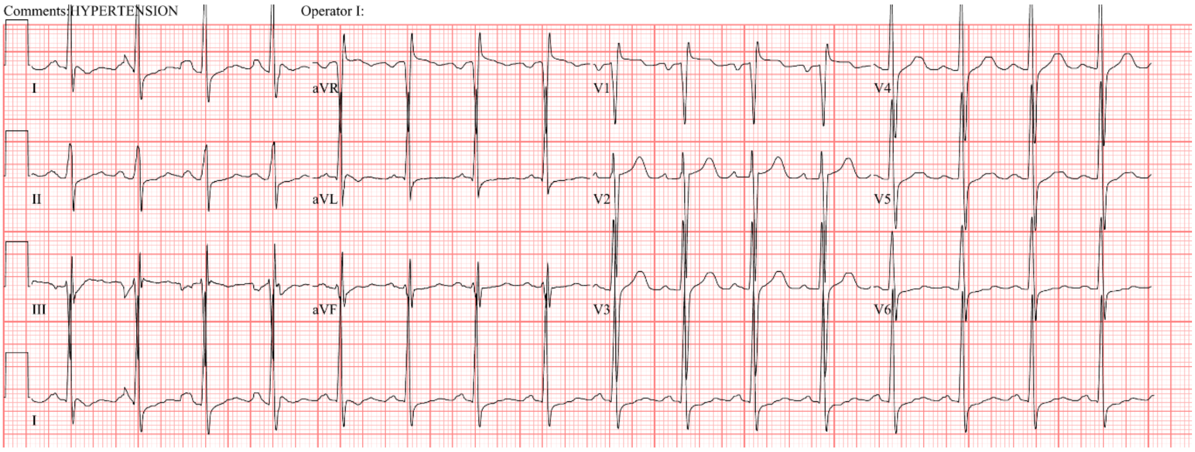

RESEARCH?: REDACTED

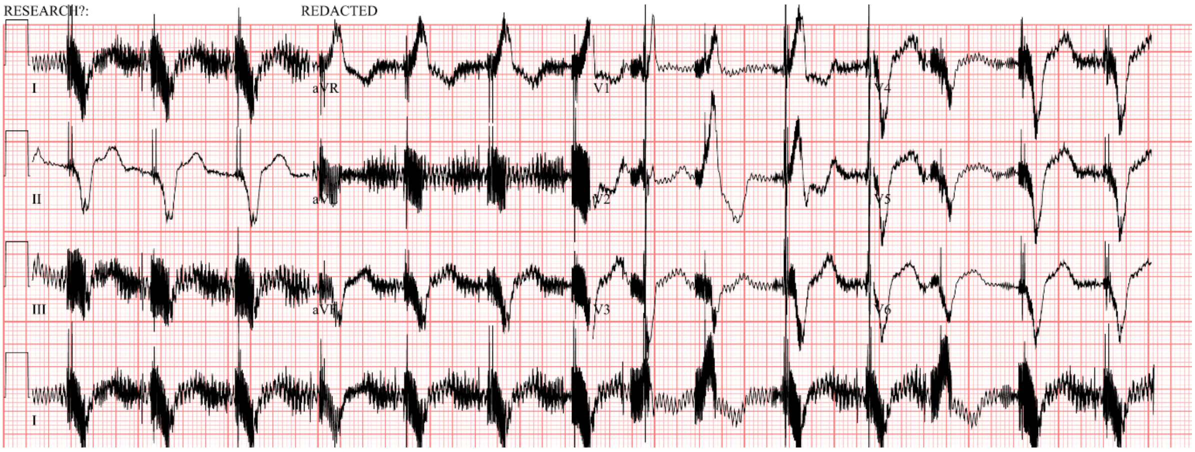

Comments:CHEST PAIN

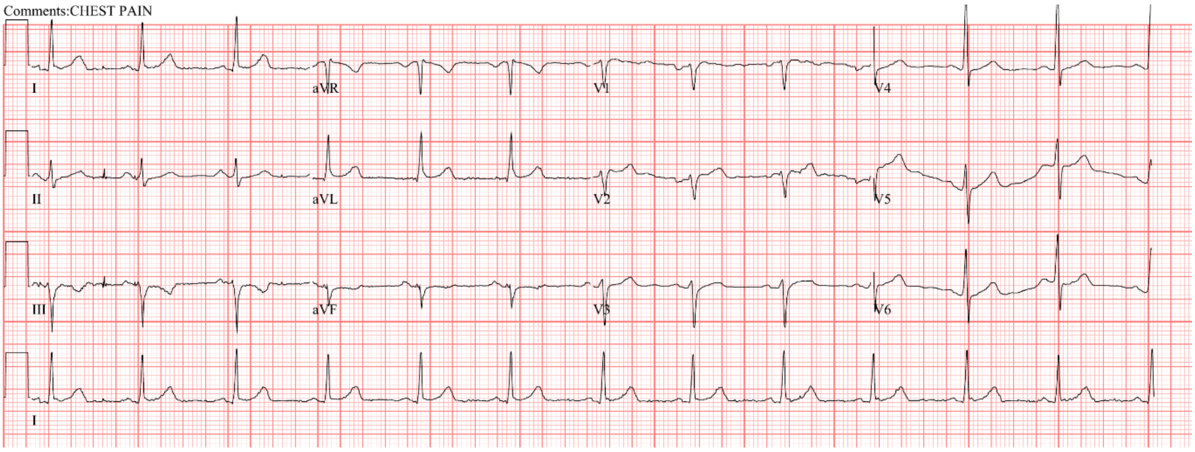

Figure S2. Failed detected 12 by 1 ECG images from 310 12 by 1 ECG images (Failure Reason: line detection failure)

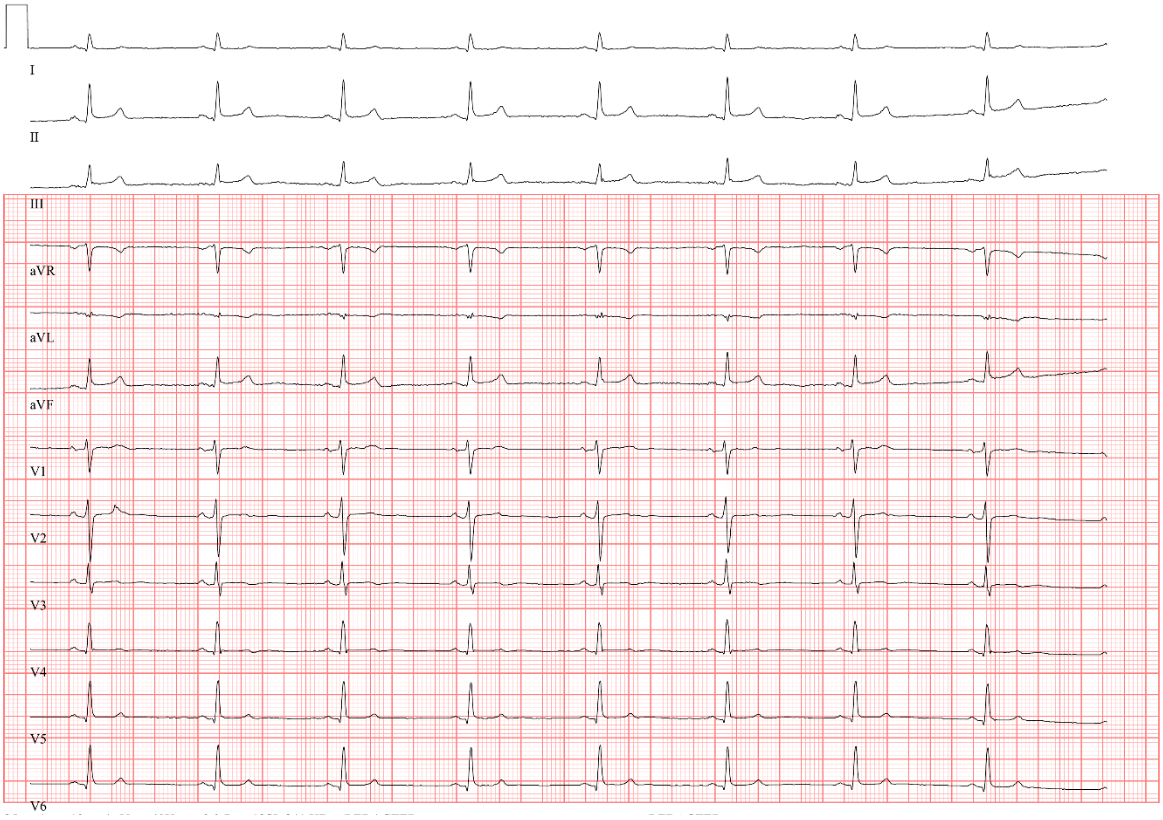

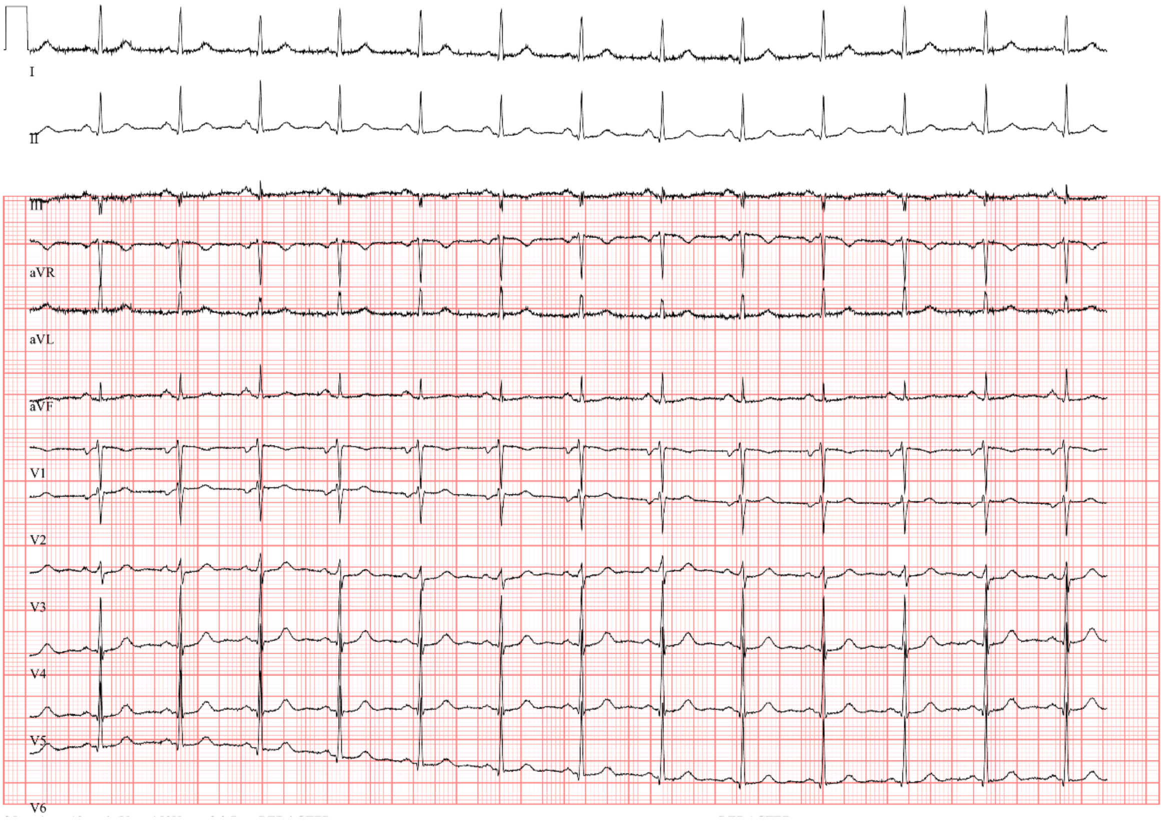

Figure S3. Failed detected 3 by 1 ECG images from 91 3 by 1 ECG images (Failure Reason: detects some parts of ECG signal as lead name II or lead name V1)

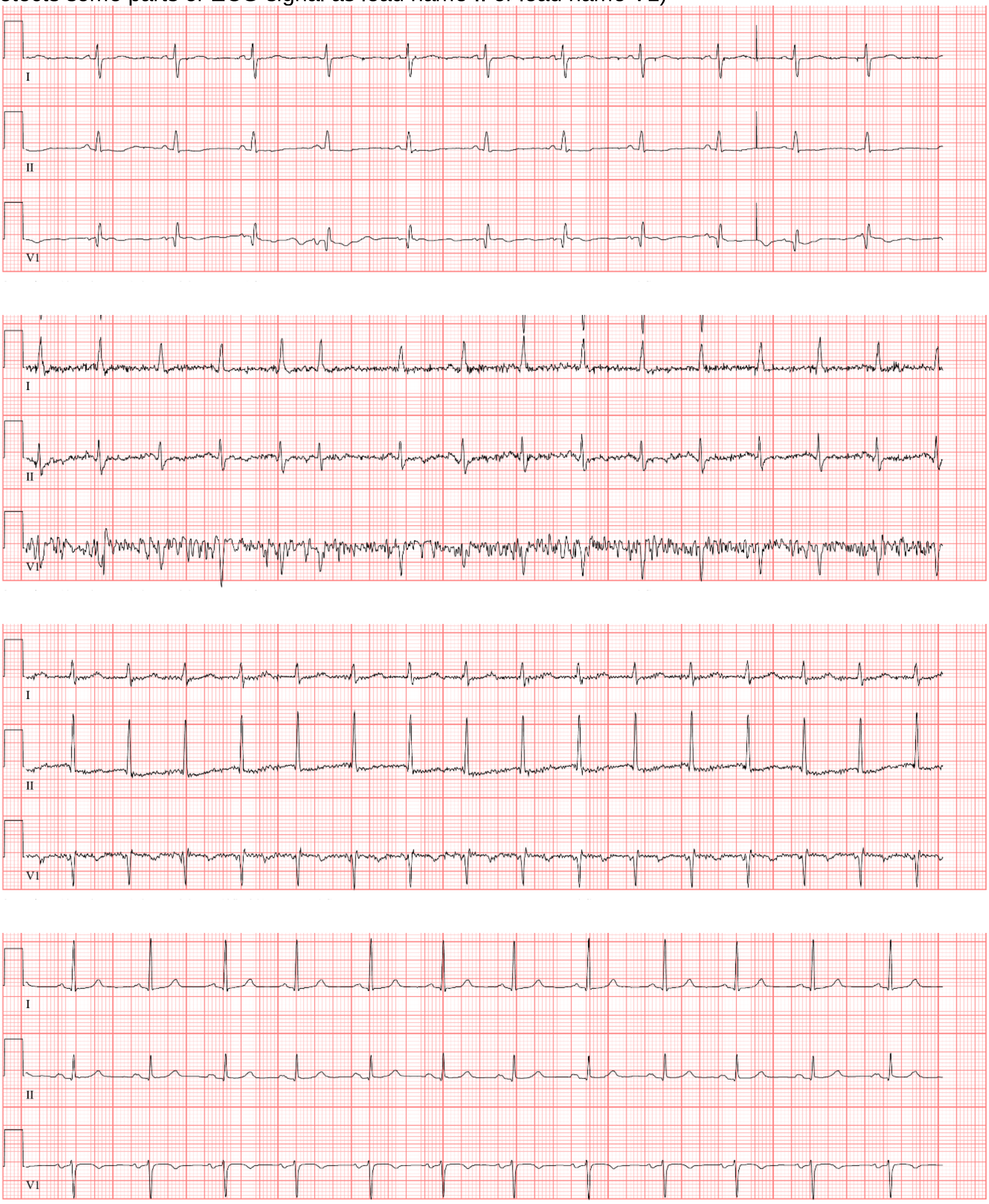

Supplement: Supplementary file 1 — Supplementary Information. [file 41598_2022_25284_MOESM1_ESM.pdf]
